# Overview of Current Therapeutics and Novel Candidates Against Influenza, Respiratory Syncytial Virus, and Middle East Respiratory Syndrome Coronavirus Infections

**DOI:** 10.3389/fmicb.2019.01327

**Published:** 2019-06-19

**Authors:** Mohammad Amin Behzadi, Victor H. Leyva-Grado

**Affiliations:** Department of Microbiology, Icahn School of Medicine at Mount Sinai, New York, NY, United States

**Keywords:** antiviral, influenza, respiratory syncytial virus, MERS-coronavirus, novel therapeutic agents

## Abstract

Emergence and re-emergence of respiratory virus infections represent a significant threat to global public health, as they occur seasonally and less frequently (such as in the case of influenza virus) as pandemic infections. Some of these viruses have been in the human population for centuries and others had recently emerged as a public health problem. Influenza viruses have been affecting the human population for a long time now; however, their ability to rapidly evolve through antigenic drift and antigenic shift causes the emergence of new strains. A recent example of these events is the avian-origin H7N9 influenza virus outbreak currently undergoing in China. Human H7N9 influenza viruses are resistant to amantadines and some strains are also resistant to neuraminidase inhibitors greatly limiting the options for treatment. Respiratory syncytial virus (RSV) may cause a lower respiratory tract infection characterized by bronchiolitis and pneumonia mainly in children and the elderly. Infection with RSV can cause severe disease and even death, imposing a severe burden for pediatric and geriatric health systems worldwide. Treatment for RSV is mainly supportive since the only approved therapy, a monoclonal antibody, is recommended for prophylactic use in high-risk patients. The Middle East respiratory syndrome coronavirus (MERS-CoV) is a newly emerging respiratory virus. The virus was first recognized in 2012 and it is associated with a lower respiratory tract disease that is more severe in patients with comorbidities. No licensed vaccines or antivirals have been yet approved for the treatment of MERS-CoV in humans. It is clear that the discovery and development of novel antivirals that can be used alone or in combination with existing therapies to treat these important respiratory viral infections are critical. In this review, we will describe some of the novel therapeutics currently under development for the treatment of these infections.

## Introduction

Respiratory viral infections are of global public health concern because they are the most common cause of symptomatic disease leading to a heavy economic burden due to an increased number of sick days (Borchardt and Rolston, 2012; Kim et al., 2017). In addition, respiratory diseases are one of the most common causes of mortality in developing countries (Ferkol and Schraufnagel, 2014). Among the two leading causes of respiratory virus infection are influenza (A and B) viruses and the respiratory syncytial virus (RSV) (Amarelle et al., 2017; Heylen et al., 2017; Yip et al., 2018). Most cases are observed in infants and children although the elderly and the immunocompromised are also at high risk of getting infected and develop severe disease (Jorquera and Tripp, 2017).

**Figure 1:**
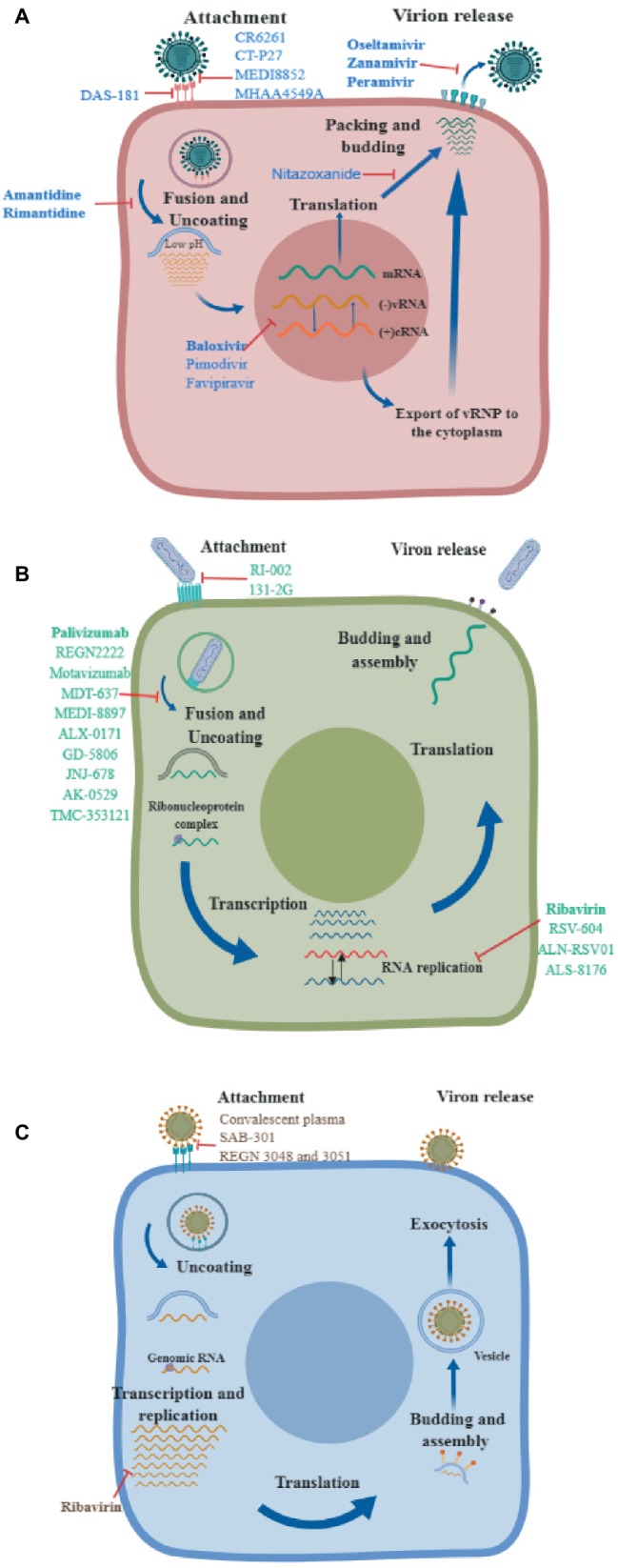
Mechanisms of action of different antivirals. A diagram of the virus life cycle of **(A)** influenza virus, **(B)** respiratory syncytial virus, and **(C)** Middle East respiratory syndrome coronavirus, indicating where each therapeutic exerts its antiviral activity. Drugs approved by the FDA are shown in bold.

While influenza viruses and RSV circulate seasonally between beginning of fall and early spring, the increased numbers of avian influenza (H7N9) in China including highly pathogenic (HP) strains of the virus, the continued outbreaks of the HP avian influenza (HPAI) H5N1 viruses, novel variants of seasonal influenza viruses (H3N2var), and the emergence of the Middle East respiratory syndrome coronavirus (MERS-CoV) highlight the serious and important need for developing novel, more effective antiviral therapies (Zumla et al., 2016; International Society for Influenza and other Respiratory Viral Diseases, 2018). This review article focuses on therapies currently under development for treatment of influenza, RSV, and MERS-CoV infections. We will briefly review drugs that have been approved by the United States Federal Drug Administration (FDA) and then drugs that are in clinical trials (Figure 1). When available, the clinical trial identifier is included.

## Influenza Virus

Influenza virus is a negative sense, segmented RNA virus that presents a substantial burden to human health. Despite the availability of successful vaccines and antivirals, infection with seasonal influenza viruses is still the cause of 3–5 million cases of severe illnesses and up to 300–650 thousand deaths worldwide (World Health Organization, 2017). In the US alone, it is estimated that seasonal influenza affects up to 9 million people a year resulting in 12,000–56,000 deaths annually (Centers for Disease Control and Prevention, 2018c). The economic burden is estimated to be in the tens of billions of dollars (Putri et al., 2018). In addition, pandemic influenza outbreaks emerge at unpredictable intervals causing increased morbidity, mortality, and a negative economic impact (Erbelding et al., 2018).

### Currently Federal Drug Administration-Approved Anti-influenza Antivirals

Briefly, these drugs can be divided in three groups: the M2 ion channel blockers, the neuraminidase inhibitors (NAIs), and the virus polymerase inhibitors. Amantadine and rimantadine are adamantane derivatives that inhibit viral replication by blocking the proton conductivity of the M2 ion channel preventing delivery of the virus ribonucleoprotein in the cytoplasm (Schnell and Chou, 2008; Ison, 2017). These compounds are specific for influenza A viruses since the M2 protein of influenza B viruses is structurally different (Wang et al., 2009; Pielak and Chou, 2011). In recent past seasons, surveillance studies reported high levels of resistance (>99%) to adamantanes among circulating influenza A(H3N2) and influenza A(H1N1)pdm09 viruses, and the centers for disease control and prevention (CDC) does not recommend these compounds for antiviral treatment or chemoprophylaxis of currently circulating influenza A viruses (Centers for Disease Control and Prevention, 2018a). Development of NAI started in the early 1970s with derivatives of 2-deoxy-2,3-dehydro-N-acetylneuraminic acid (Palese et al., 1970), although it was not until 1999 that the first NAI was approved for use in humans (Chaudhuri et al., 2018). The NAI binds to the neuraminidase of the virus and prevents its cleavage, thus inhibiting the release of new virus particles (Ison, 2017). NAIs currently approved by the FDA include: oseltamivir, zanamivir, and peramivir. Oseltamivir acid is a pro-drug indicated for oral administration. Once in the gastrointestinal tract, the pro-dug is rapidly cleaved to the active metabolite, oseltamivir carboxylate (Kim et al., 2017). Due to poor oral bioavailability, zanamivir is approved for inhalation delivery with intravenous administration only available by compassionate use (Ison, 2017; Shaw, 2017). Peramivir also has poor oral bioavailability; therefore, it has been approved for intravenous administration only. Because the drug reaches high concentrations in plasma and respiratory tissues, it has been approved as a single-dose infusion. One of the inherit problems of these drugs is that, in order to be effective, treatment should start within 48 h post-exposure. Antiviral resistance to oseltamivir, zanamivir, and peramivir is very low in currently circulating influenza virus strains, although this can change because several clusters of oseltamivir resistance have been detected in Japan, Australia, and China (Takashita et al., 2015; Alame et al., 2016; Koszalka et al., 2017). Also known as S-033188, baloxavir marboxil (Xofluza®) is a pro-drug that is hydrolyzed *in vivo* to S-033447, the active form that selectively inhibits cap-dependent endonuclease, preventing the initiation of mRNA synthesis of the influenza virus (Takashita et al., 2018). This is a potent small molecule that shows activity against several influenza A viruses, including oseltamivir-resistant viruses as well as B viruses (Noshi et al., 2018). Preclinical studies demonstrated that treated mice infected with influenza virus were protected from clinical signs and mortality even in a delay of treatment approach (treatment started 4 days post-infection). Furthermore, a subtherapeutic dose of baloxavir in combination with oseltamivir also protected mice from infection and mortality (Fukao et al., 2018). In addition, studies in mice infected with avian influenza viruses such as H5N1 or H7N9 also demonstrated protection after oral administration with baloxavir (Uehara et al., 2016). A clinical study (NCT02954354) aimed to compare the efficacy of baloxavir with a placebo or oseltamivir in healthy patients infected with influenza demonstrated that the drug was well tolerated and was associated with a significant reduction in viral load compared to the oseltamivir group. Time of alleviation of symptoms was similar to oseltamivir. The currently undergoing clinical program for this drug includes phase 3 clinical trials to determine safety, pharmacokinetics, and efficacy in healthy pediatric participants aged less than 1 year (NCT03653364) or in pediatric patients with influenza-like symptoms (NCT03629184) and a study to assess efficacy and safety of baloxavir in combination with standard-of-care neuraminidase inhibitor in hospitalized participants with severe influenza (NCT03684044). These studies are currently recruiting and expected to be concluded in spring 2020. In Japan, baloxavir has been approved for the treatment of adult and infant patients infected with influenza; while in the US, the drug has just been approved by the FDA for the treatment of acute, uncomplicated influenza in people aged 12 years and older (Food and Drug Administration, 2018). The emergence of resistant variants to polymerase inhibitors has been observed and it is conferred by an I38T mutation in the PA polymerase (Jones et al., 2018). In the same study, a novel mutation conferring resistance (E23K) was also observed. Both mutations have been encountered during clinical trials for baloxavir (Hayden et al., 2018).

### Promising Drug Candidates in the Pipeline

Given the inherit limitations of these currently approved compounds and the potential risk for the arising of antiviral resistance, there is still an urgent need for developing new anti-influenza drugs. These novel drugs should have some (ideally all) of the following characteristics: effective when delivered late in infection, low propensity for developing antiviral resistance, broad activity (influenza A and B), improved effectiveness compared to the standard of care, and can be easily administered in uncomplicated as well as complicated cases of influenza (Koszalka et al., 2017; Shaw, 2017). Next, we will summarize the most advanced (phase 2 and 3 clinical trials), promising drug candidates.

## Viral Targeting Candidates

### Antibodies

New and more efficient technologies for the production of monoclonal antibodies (mAbs) have stimulated the development of novel mAbs-based therapies for influenza and other infectious diseases (Jin et al., 2017). A number of broadly neutralizing mAbs targeting the conserved stalk region of the influenza virus hemagglutinin (HA) molecule are currently under development. In addition, a non-neutralizing mAb targeting the virus matrix protein is also in clinical trials.

#### CR6261 and CR8020

CR6261 is directed against a highly conserved helical region in the membrane-proximal stalk of hemagglutinin. *In vitro* studies on this class of mAbs demonstrated neutralization activity across a broad spectrum of influenza A subtypes. CR6261 is protective in mice against lethal doses of H1N1 and H5N1 viruses (Koudstaal et al., 2009). A challenge study successfully demonstrated both therapeutic and prophylactic efficacy in the ferret model against a lethal dose of an H5N1 virus (Friesen et al., 2010). A clinical trial phase 2 (NCT02371668) aimed to determine if the CR6261 reduces flu disease in people treated with this drug versus a placebo has concluded but results are still not available. CR8020 targets an immunosubdominant, relatively conserved membrane-proximal stalk region of hemagglutinin. The mAb is active against group 2 influenza viruses and has been shown to have neutralization activity against H3, H7, and H10 subtypes both *in vitro* and *in vivo* (Ekiert et al., 2011). A phase 2a trial to evaluate the protective efficacy and safety of CR8020 in an influenza challenge model was completed in 2014 (NCT01938352). Unfortunately, a recent study showed that CR8020 targets a region of the HA stalk that is prone to escape, therefore escape mutants are likely to arise (Tharakaraman et al., 2014).

#### CT-P27

This product contains two distinct but complementary mAbs that have shown broad efficacy *in vitro* and *in vivo* against different group 1 and group 2 influenza viruses. A phase 2a clinical trial (NCT02071914) was conducted to evaluate the efficacy and safety of CT-P27 in an influenza challenge model. The study concluded in July 2014, however no results were reported. Currently recruiting, a phase 2b clinical trial (NCT03511066) is aimed to evaluate the efficacy and safety of the mAbs combo in comparison to placebo in subjects with acute uncomplicated influenza A infection.

#### MEDI8852

This is a potent, broadly neutralizing investigational human IgG1 mAb targeting a highly conserved stalk region of the HA protein. MEDI8852 has been reported to effectively neutralize all known influenza A HA subtypes (Kallewaard et al., 2016). Preclinical studies in animal models have shown efficacy in protecting against H5N1 influenza virus lethal challenges when used alone or in combination with oseltamivir (Paules et al., 2017). A phase 2a study (NCT02603952) to evaluate the safety of MEDI8852 in adults with acute, uncomplicated influenza was recently concluded and results demonstrated an acceptable safety profile in this group of patients. In 2016, MEDI8852 received Fast Track designation by the FDA that allows the drug an expedite development and review (Ali et al., 2018).

#### MHAA4549A

This is a human monoclonal IgG1 antibody containing a VH 3-30 59 heavy chain paired with a Vκ1-15 light chain (McBride et al., 2017). MHAA4549A targets a highly conserved epitope on the stalk region of HA and is capable of neutralizing all tested seasonal human influenza A strains (Nakamura et al., 2013). Two mechanisms of action have been described for this mAb, one by binding the HA to avid virus infectivity and two by antibody-dependent cell-mediated cytotoxicity to increase killing of infected cells (McBride et al., 2017). Preclinical studies demonstrated efficacy in the mouse model against a diverse group of influenza viruses including group 1 and group 2 strains even when administered 72 h post-infection. In addition, synergism was observed when administered with oseltamivir at 48 h post-infection. Finally, studies in ferrets demonstrated protection against an H5N1 HPAI virus challenge (Nakamura et al., 2013). A few clinical trials have been conducted to evaluate safety, pharmacokinetics, and efficacy of MHAA4549A alone or in combination with oseltamivir (Lim et al., 2016; McBride et al., 2017; Deng et al., 2018). Results established that the mAb is well tolerated with a half-life similar to other human antibodies (~23 days) but with a nonlinear nasal pharmacokinetics (McBride et al., 2017; Deng et al., 2018). Furthermore, treatment significantly reduced viral loads (McBride et al., 2017). The most recent clinical trial (NCT02293863) aimed to investigate the safety and clinical activity of a single intravenous (IV) dose of MHAA4549A in adult participants hospitalized with severe influenza A in combination with oseltamivir was recently concluded and results indicated no advantage on any of the primary clinical outcomes evaluated when compared with the standard of care. Furthermore, no significant differences were observed in any of the virologic outcomes evaluated including viral shedding or peak of virus load.

### Virus Polymerase Inhibitors

#### Pimodivir

This is a novel, non-nucleoside polymerase inhibitor that targets the PB2 subunit of influenza A viruses. Targeting the PB2 avoids docking of the 7-methyl GTP cap structure, thus preventing viral RNA synthesis (Clark et al., 2014; Boyd et al., 2015). The early activity of pimovidir in the cell cycle has shown to improve cell viability compared to oseltamivir (Byrn et al., 2015). Furthermore, it is active against a diverse group of influenza A viruses including the H1N1pdm, H5N1, and H7N9 (Byrn et al., 2015). Preclinical studies in mice demonstrated protection against a lethal challenge even when given 4 days post-infection with H1N1pdm or H5N1 viruses (Byrn et al., 2015). Further studies also showed protection against a lethal challenge using an H3N2 virus (Smee et al., 2016). Studies using pimodivir and oseltamivir suggest a potential benefit for a combination therapy (Trevejo et al., 2018). A phase 2b clinical study (NCT02342249) treatment with pimodivir significantly decreased viral load over 7 days versus placebo, in adult patients with acute, uncomplicated seasonal influenza A. Recently, pimodivir has received FDA Fast Track designation due to its potential to address an unmet medical need in those who develop influenza A infection complications. A phase 3 clinical trial (NCT03381196) aimed to evaluate the efficacy and safety of pimodivir in combination with oseltamivir in adults at risk of developing complications is currently recruiting patients and it is expected to be concluded in October 2019.

#### Favipiravir

Formerly known as T-705, this substituted pyrazinecarboxamide derivative is an inhibitor of the influenza virus polymerase. As a purine nucleoside analog, favipiravir directly inhibits the activity of the RNA-dependent RNA polymerase (Furuta et al., 2005) and it has broad-spectrum activity against all influenza subtypes including those resistant to neuraminidase and M2 inhibitors (Furuta et al., 2017). Preclinical studies demonstrated the broad antiviral spectrum of this compound against different human and avian influenza A and B viruses including avian influenza viruses H5N1 and H7N9 (Sidwell et al., 2007; Watanabe et al., 2013) where it has shown protection and reduction of virus titers in the infected mice. Furthermore, mice treated with favipiravir had a better outcome after an H7N9 infection compared to mice treated with neuraminidase inhibitors (Watanabe et al., 2013). Combination therapy studies found that this drug works synergistically with neuraminidase inhibitors to improve lung virus titers, body weight loss, and survival in mice infected with a pandemic H1N1 influenza virus (Tarbet et al., 2012) or with an avian influenza virus H5N1 (Smee et al., 2010). Favipiravir is approved in Japan, but its use is restricted to patients infected with an influenza virus resistant to NAI or in the event of a pandemic (Toyama Chemical Co. Ltd). These conditions have been put in place due to concerns of teratogenicity, which have been identified in animal experiments. A couple of phase 3 clinical trials aimed to determine the efficacy and safety of favipiravir for the treatment of uncomplicated influenza infection in adults have been completed but the results have not been published yet. A new phase 2a clinical trial study to determine the pharmacokinetics of favipiravir in patients with severe influenza is reported as active but not currently recruiting with estimated study completion date of March 2019 (NCT03394209). This is a very promising broad-spectrum anti-influenza antiviral that also has a broad antiviral activity against several other RNA viruses (Davidson, 2018). A couple of caveats may limit the use of this antiviral to compassionate use or in the case of emerging or re-emerging viruses for which a treatment is not available. Favipiravir has the potential for teratogenicity and embryotoxicity and is the reason why it has conditional approval in Japan (Furuta et al., 2017). A study of samples obtained for a clinical trial tested the emergence of mutations in viruses obtained from paired patients before and 1 or 2 days after the initiation of treatment showed the emergence of amino acid substitutions in the RNA polymerase subunits. Although these mutations did not significantly reduce the susceptibility to favipiravir (Takashita et al., 2016)*, in vitro* escalation studies showed that influenza viruses can adapt to low concentration of the compound which highlights the potential for some influenza virus strains to possibly develop resistance (Ormond et al., 2017).

## Host-Targeting Candidates

### Fludase

Also known as DAS-181, this is a recombinant sialidase composed of the catalytic domain of a bacterial sialidase (*Actinomyces* *viscosus*) and the epithelium-anchoring domain of the human protein amphiregulin (Malakhov et al., 2006). In the human respiratory tract, cell surface sialic acids are the primary receptors for binding and entry of influenza viruses. Fludase works by removing sialic receptors from the airway epithelium, therefore preventing viral entry into cells of the respiratory epithelium (Triana-Baltzer et al., 2011). Preclinical *in vitro* studies show that fludase is a broad-spectrum anti-influenza antiviral with potent antiviral activity against human and avian influenza viruses with no cellular toxicity (Triana-Baltzer et al., 2009, 2010). *In vivo* studies showed that mice can be rescued from influenza infection after treatment with fludase, including after infection with highly pathogenic H5N1 and H7N9 viruses (Belser et al., 2007; Marjuki et al., 2014, 2015). In a randomized, double-blind, placebo-controlled phase 2 study conducted to determine the effective dose in healthy adult participants with laboratory-confirmed influenza (NCT01037205), it was observed that treatment with fludase significantly reduces viral load and viral shedding after multiple doses. In addition, no significant adverse effects were observed in the patients that received the treatment (Moss et al., 2012). In a phase 1 clinical trial (NCT01651494), it was observed that in longer treatments (more than 7 days), patients developed antibodies against the compound leading to a reduced efficacy (Zenilman et al., 2015).

### Nitazoxanide (Alina^®^)

Nitazoxanide (NTZ) was originally developed and licensed as an antiprotozoal drug for the treatment of enteritis caused by *Cryptosporidium* and *Giardia* infections. Nitazoxanide is a thiazolide compound that is rapidly deacetylated in the blood to the active metabolic form tizoxanide (Rossignol, 2009). In addition to its antiparasitic activity, this compound has shown activity against a broad range of viruses including influenza viruses (Belardo et al., 2015; Tilmanis et al., 2017; Stachulski et al., 2018). The mechanism of action against influenza virus is achieved by impairing the trafficking of the viral hemagglutinin (HA) from the endoplasmic reticulum to the Golgi apparatus and by blocking HA terminal glycosylation leading to block maturation of the HA (Rossignol et al., 2009). Preclinical studies demonstrated an antiviral activity against different strains of influenza A, and B viruses *in vitro* including avian influenza viruses (Sleeman et al., 2014). There are not published studies of the antiviral efficacy of NTZ in animal models, although extensive pharmacological testing has been performed in animals (Koszalka et al., 2017). Nitazoxanide is an example of a repurposed drug with a lot of studies in humans, which is facilitating the advancement of this drug as a treatment for influenza virus infection (Rossignol, 2009). A phase 2b/3 clinical trial (NCT01227421) aimed to determine the safety and efficacy of NTZ in the treatment of acute uncomplicated influenza established that treatment with 300 mg twice a day for 5 days was well tolerated and it was associated with a reduction of symptoms and infectious viral load (Haffizulla et al., 2014). A phase 3 study (NCT03336619) to evaluate the efficacy and safety of 300-mg nitazoxanide tablets (Stachulski et al., 2018) in the treatment of uncomplicated influenza was concluded in March 2019 but results have not been posted.

## Respiratory Syncytial Virus

Respiratory syncytial virus is the most commonly identified cause of acute lower respiratory tract infection in infants, children, immunocompromised adults, and the elderly (Zorc and Hall, 2010; Griffiths et al., 2017). Since the first isolation of the virus from chimpanzees in 1956 (Morris et al., 1956), extensive research has been conducted in epidemiology, diagnosis, and animal models for the infection, but there is no vaccine and only two approved antivirals available against the virus (Xing and Proesmans, 2019).

RSV is a non-segmented virus with negative sense single-stranded RNA genome encoding 11 proteins. Out of those, two surface transmembrane proteins, G and F, have been shown to play a key role in RSV binding and fusion, respectively. The virus is classified into two subtypes: A and B, with about 50% genetic diversity in the G gene and 10% differences in the F gene (Tian et al., 2017; Xing and Proesmans, 2019). Due to less antigenic variability of the F protein compared with the G protein, this protein is the main target of research for developing antivirals as well as anti-RSV vaccines (Taleb et al., 2018).

### Currently Federal Drug Administration-Approved Anti-Respiratory Syncytial Virus Antivirals

There are only two RSV antiviral drugs approved by the FDA for the treatment or prevention of serious respiratory tract infections caused by RSV: aerosolized ribavirin for treatment and palivizumab (Synagis^®^) for prophylaxis.

The guanosine analog ribavirin is a broad-spectrum antiviral agent with activity against RSV and other RNA viruses such as hepatitis C and Zika viruses (Antonini et al., 2018; Kim et al., 2018; Zhurilo et al., 2018). The beneficial effect of this drug in inhibiting RSV replication was demonstrated in several studies. Ribavirin showed antiviral activity against RSV and reduced RSV lung titers in infected cotton rats (Bonavia et al., 2011). Similarly, significant clinical benefits have been observed in children treated with aerosolized ribavirin early in infection (Devincenzo, 2000). However, the clinical application of ribavirin is limited because of its nonspecific anti-RSV activity, risks for potential toxicity, and relatively high cost (Sun et al., 2013).

Palivizumab is a humanized monoclonal antibody found to have strong RSV-neutralizing capability against a range of RSV strains. The palivizumab 24-aa epitope structure on the RSV F protein is well characterized (Schickli et al., 2015). The drug has been shown to be effective in preventing RSV-related hospitalizations in children (Frogel et al., 2008). Although the efficacy of this antiviral against RSV infection is proven, due to the considerable cost of palivizumab, the usage is limited to the high-risk populations (Shahabi et al., 2017) including infants and young children (Centers for Disease Control and Prevention, 2018b).

## Promising New Antiviral Candidates in the Pipeline

In recent years, in addition to the FDA-approved RSV antivirals, a number of alternative strategies against RSV including new recombinant antibodies, nanobodies, small molecules such as fusion inhibitors, nucleoprotein inhibitors, nucleoside analogs, and non-nucleoside inhibitors are being developed.

### Antibodies

Intravenous polyclonal immunoglobulin (IVIG) is a preparation of human serum that contains high titers of neutralizing antibodies against the virus. Usage of IVIG in severe RSV infections in high-risk children significantly reduced the rate of hospitalizations due to related respiratory infections (The PREVENT Study Group, 1997). Currently, new recombinant mAbs against RSV are in different stages of development.

#### RI-001 and RI-002

These are aqueous intravenous polyclonal human immunoglobulin G (IgG) from pools of source plasma of screened healthy adult donors with high levels of RSV-neutralizing antibodies. The results of a phase 1 and a 2 study on RI-001 and RI-002, respectively, demonstrated that the preparation met acceptable pharmacokinetic and safety criteria, and achieved a fold-change increase in titer of anti-RSV neutralizing antibodies in patients (Wasserman et al., 2017).

#### REGN2222

REGN2222 is a fully human IgG1 mAb produced in VelocImmune mice that binds specifically to the F protein of RSV. Preclinical studies in cotton rats demonstrated that REGN2222 is effective at reducing RSV viral replication in the lungs (Gurnett-Bander et al., 2016). Although the results of two clinical trials in phase 1 in healthy adults showed that the antibody was well tolerated and it had a half-life longer than typical IgG1 mAbs with low immunogenicity (Sivapalasingam et al., 2015), the results of a recent phase 3 trial in preterm infants revealed that this mAb did not meet its primary endpoint of preventing RSV.

#### MEDI8897

MEDI8897 is a recombinant human IgG1κ mAb with an engineered Fc region to have a longer serum half-life and designed for prevention of lower respiratory tract illness (LRTI) caused by RSV. The antibody binds to the prefusion conformation of the F protein of RSV. The results of placebo-controlled studies in healthy adults and healthy preterm infants showed an increase in the mean half-life of MEDI8897. Moreover, the safety profile of MEDI8897 was similar to placebo (Griffin et al., 2017; Domachowske et al., 2018). To evaluate the efficacy of this mAb in preventing infection, further clinical studies are still needed in the target population of infants entering their first RSV season.

#### ALX-0171

ALX-0171, the first nanobody developed for treatment of RSV infection, is a trivalent nanobody (42 kDa) that binds the antigenic site II of the RSV F protein and neutralizes both subtypes of RSV. *In vitro* neutralization efficiency of ALX-0171 is greater than palivizumab (Detalle et al., 2016). Preclinical studies in cotton rats and neonatal lambs have indicated that ALX-0171 is highly effective in reducing both nasal and lung RSV titers (Hanton et al., 2012; Detalle et al., 2014). The results of phase 1 and 2 clinical studies demonstrated that inhaled ALX-0171 inhibited RSV replication and reduced viral load compared to placebo. ALX-0171 is currently in clinical trials (NCT03418571) to treat infants hospitalized with RSV infection (Van Heeke et al., 2017).

#### mAb 131-2G

131-2G is a murine mAb that binds to the central conserved region of the RSV G protein and interferes with the attachment process by blocking the G protein from binding to CX3CR1 (Johnson et al., 2015). RSV-challenged mice studies showed that early treatment with this mAb reduced both pulmonary inflammation and lung virus titers (Haynes et al., 2009). Clinical studies are needed to evaluate the safety and efficacy of this antiviral in humans.

#### Motavizumab

Motavizumab is a recombinant humanized mAb that binds to a 24-residue, linear, conformational epitope FFL on the RSV F glycoprotein (Zhu et al., 2011). Preclinical studies in cotton rats demonstrated that the mAb decreased RSV titers more than 50 times in lungs in comparison to palivizumab (Wu et al., 2007). Phase 1 and 2 clinical trials showed that motavizumab is well tolerated in healthy adults and premature infants as well as in high-risk children with chronic lung disease of prematurity (Abarca et al., 2009; O’Brien et al., 2015). The results of a recent phase 3 clinical trial to prevent serious RSV disease in healthy term infants indicated that motavizumab reduced the proportion of infants admitted to the hospital with RSV (O’Brien et al., 2015).

### Fusion Inhibitors (Small Molecules)

Numerous studies have evaluated the virus-host interactions to determine the viral and host factors that contribute to replication of viruses. For RSV replication, both the co-receptors that become localized on the epithelium in response to virus-mediated signals and the RSV F protein contribute to this process (Hotard et al., 2015). Most antiviral small molecules that bind to the F protein can block membrane fusion and viral penetration, and subsequently inhibit RSV replication (Battles et al., 2016).

#### GS-5806

GS-5806 is an orally bioavailable RSV fusion inhibitor that has been shown to prevent RSV entry by blocking the virus-cell fusion process (Samuel et al., 2015). In a preclinical study conducted in cotton rats, an efficient bioavailability and penetration of the drug in lung tissue were observed. The results of a clinical study in healthy volunteers experimentally infected with RSV indicated that oral administration of GS-5806 resulted in reduction of viral load and disease severity (Mackman et al., 2015). More clinical trials have been completed in different populations including in RSV-infected hospitalized patients, lung transplant recipients, and in hematopoietic stem cell transplantation recipients, but the results are not available yet. Recently published results for phase 2b trials in hospitalized adults with RSV did not show significant reduction in viral load or improvement of clinical outcomes to the disease (Hanfelt-Goade et al., 2018). To clarify the efficacy of this drug, further studies are required in infants and children.

#### MDT-637

MDT-637 (VP-14637) is a fusion inhibitor that has been shown to inhibit RSV entry to the cells. Both *in vitro* reports and preclinical studies in cotton rats demonstrated the anti-RSV activity of MDT-637. This compound is effective against both subtypes of RSV (Kim et al., 2017). The reformulated form of MDT-637, VP-14637, can be administered by powder inhaler with a quick delivery to the respiratory tract (Wyde et al., 2005). The results of phase 1 clinical trials indicated a safety and desirable pharmacokinetic profile of the drug in healthy adults (NCT01475305) (Kim et al., 2017).

#### JNJ-53718678

JNJ-678 is a potent RSV-specific fusion inhibitor that has been shown to act as an effective anti-RSV *in vitro* and in animal models (Roymans et al., 2014; Ackermann et al., 2016). Clinical studies in healthy adults assessed its antiviral efficacy as well as safety. It was shown that JNJ-53718678 reduced RSV viral load, respiratory infections’ severity caused by RSV, and duration of the disease (Israel et al., 2016; Huntjens et al., 2017). Further studies are needed in infants and children to prove the antiviral activity of this anti-RSV candidate.

#### AK-0529

AK-0529 is a novel compound being developed to inhibit RSV replication by blocking viral entry into the target cells. A placebo-controlled phase 1 clinical trial showed that oral administration of this antiviral is well tolerated among healthy volunteers and meets the standard safety profile (Toovey et al., 2015). A phase 2 clinical trial in hospitalized infants infected with RSV is currently underway (NCT02654171).

#### TMC353121

TMC353121, a small substituted benzimidazole RSV fusion inhibitor, is an improved derivative of JNJ-2408068 [a compound with antiviral activity against both RSV A and B, but with long tissue retention times in animal models which created concerns and stopped its further development (Wyde et al., 2003; Bonfanti et al., 2007)].TMC353121, in contrast to JNJ-2408068, lacks the long tissue retention but maintains its high antiviral activity *in vitro* (Bonfanti et al., 2008). Using TMC353121 in preclinical studies in cotton rats (Rouan et al., 2010) and African green monkeys (Ispas et al., 2015) revealed a reduction in viral loads in a dose-dependent manner and in lung inflammation. Based on these data, TMC353121 could be a drug candidate for treatment of RSV infection; however, safety studies and more clinical evaluations are still needed.

### Nucleoprotein Inhibitors

One promising area of development revolves around inhibitors of RSV that target the virus nucleoprotein. RSV nucleoprotein is involved in virus assembly and it is crucial for the virus replication. Less genetic variation of this protein among RSV A and B subtypes makes it an amenable molecular target for development of anti-RSV compound (Collins et al., 2013).

#### RSV-604

RSV604 is a small molecule with submicromolar RSV activity that was discovered through chemical optimization of an RSV high-throughput screen hit (Chapman et al., 2007). It is effective against both RSV A and B replication at a post-entry step. The antiviral mechanism of this compound is related to its binding to the RSV N protein and therefore inhibition of both RNA synthesis and the infectivity of released virus (Challa et al., 2015). A phase 2a clinical trial in stem cell transplant patients with RSV infection showed that following treatment with RSV604, plasma exposure reached the 90% effective concentration (EC_90_). In addition, patients had a reduction in viral titers and RSV infection symptoms (Marty et al., 2007).

#### ALN-RSV01

ALN-RSV01 is a small interfering RNA (siRNA) with 19 nucleotides targeting a highly conserved region of the RSV nucleoprotein gene. This siRNA has shown to be effective in both prophylaxis and treatment models. Results of a preclinical study in mice indicate that treatment with ALN-RSV01 achieved up to 3-logs reduction in RSV lung titers in comparison to control groups (Lu et al., 2018). Intranasal administration of ALN-RSV01 in healthy volunteers inoculated with RSV showed a significant reduction of RSV infection in active drug recipients when compared to the placebo recipients group (DeVincenzo et al., 2010). In a phase 2 clinical trial conducted in lung transplant recipients with RSV infection, a good safety profile and positive benefits of this siRNA were observed in reducing the risk of bronchiolitis obliterans syndrome (Gottlieb et al., 2016). Further evaluations of this antiviral in naturally RSV-infected children and infants should be established.

### Nucleoside Analog and Non-nucleoside Inhibitors

#### ALS-008176

ALS-008176 (4 chloromethyl-2-deoxy-3,5-di-O-isobutyryl-2-fluorocytidine) is a nucleoside RSV polymerase inhibitor with a high level of oral bioavailability. A preclinical study in African green monkeys infected with RSV and treated orally demonstrated an efficient antiviral activity (Deval et al., 2015). A clinical trial in healthy adults experimentally infected with RSV revealed shorter RSV RNA clearance time in the ALS-008176 treated group compared with placebo recipients, and the reduction in RSV viral load ranged from 73 to 88% in nasal washes of ALS-008176 treated individuals (DeVincenzo et al., 2015).

## Middle East Respiratory Syndrome

MERS is an emerging zoonotic disease caused by a virus called MERS coronavirus (MERS-CoV). This virus is an enveloped, single-stranded, positive sense RNA virus with a large genome (approximately 30 kb in length) belonging to the C lineage of *Betacoronavirus* (Enjuanes et al., 2016; Wernery et al., 2017).

The disease was first reported in 2012 in the Middle East and since then multiple introductions to the human population have occurred from the dromedary, the only known animal reservoir of MERS-CoV (Wernery et al., 2017; Jordan et al., 2018; Paden et al., 2018). The majority of the 2,374 cases reported up to date (World Health Organization, 2019) have occurred in Saudi Arabia (1983 cases). The disease has been reported in 27 countries from different continents with most of them associated with recent travel to the Arabic peninsula (Park et al., 2019). Person-to-person transmission has been well documented especially in health care workers and family members and may lead to large outbreaks with a significant impact on public health as observed in the Middle East and Korea (Ki, 2015; Mo and Fisher, 2016). People from any age can be infected with the virus, although the majority of cases have been observed in adults (Centers for Disease Control and Prevention, 2017). Clinical features range from asymptomatic or mild to severe disease and death (Zumla et al., 2015; Rabaan, 2017). Symptoms may include respiratory illness that can lead to acute respiratory distress syndrome (Assiri et al., 2013; Memish et al., 2013) and digestive symptoms including nausea, vomiting, and diarrhea (Centers for Disease Control and Prevention, 2017) with some patients developing renal failure (Bermingham et al., 2012; Jordan et al., 2018). The case fatality rate worldwide is 34.6%, while in Saudi Arabia it is 37.5% (World Health Organization, 2019).

To date, there are no licensed vaccines or therapeutics for prevention or treatment of MERS-CoV infection and therapy is focused on supportive care to relive symptoms and in more severe cases to also support function of vital organs (Modjarrad, 2016; Mustafa et al., 2018). Since no specific treatment is available, supportive therapy and the use of broad-spectrum antivirals are currently the options for treatment (Rabaan et al., 2017). A broad range of therapeutics have been used in the clinic to treat MERS-CoV-infected patients, and their use is based on knowledge obtained during the severe acute respiratory syndrome (SARS) outbreak in 2003 and the recent influenza pandemic in 2009 (Mo and Fisher, 2016). Furthermore, comprehensive clinical studies are lacking and only field data are available (Dyall et al., 2017).

Therapies that have been used for treatment of patients infected with MERS-CoV include convalescent sera, corticosteroids, and/or antiviral therapeutics including interferons, ribavirin, and protease inhibitors (or combination of members from these groups)(Al-Tawfiq et al., 2014; Petersen et al., 2015; Zumla et al., 2016; Al-Tawfiq and Memish, 2017; Mustafa et al., 2018). There is a long list of potential therapeutics in preclinical phase that have shown promising results *in vitro* and in animal models. Comprehensive reviews of the current therapeutics under development have been published elsewhere (Zumla et al., 2016; Dyall et al., 2017; Rabaan et al., 2017).

### Convalescent Plasma

The use of plasma obtained from recovering patients (convalescent plasma) has shown beneficial effects in outbreaks of SARS and influenza virus infections (Al-Tawfiq et al., 2017). Studies in these infections indicated that the use of CP can reduce viral loads and mortality (Mair-Jenkins et al., 2015; Wu et al., 2015). Due to the lack of clinical data demonstrating efficacy in MERS-CoV infection, the use of CP is considered an investigational treatment by the WHO (World Health Organization, 2014). Only two cases have been reported to use intravenous immunoglobulin as part of an integral treatment intervention; therefore, a beneficial effect was not readily quantifiable (Kapoor et al., 2014; Arabi et al., 2015). In preclinical studies, passive transfer of sera from MERS-CoV immune camels to infected mice reduced weight loss and lung histopathology (Zhao et al., 2015). A protocol to determine the safety and effectiveness of CP therapy for patients infected with MERS-CoV was recently published (Arabi et al., 2015). In this study, the authors concluded that due to the variation in the levels of MERS-CoV-specific immunoglobulins in recovered patients, a large screening will be necessary to obtain sufficient plasma donors. A clinical study was registered based on these findings, but since then, it has been withdrawn (NCT02190799). The use of CP remains as a potential therapeutic intervention but the lack of evidence to prove its safety and efficacy in treating infected patients limits its potential impact (Mustafa et al., 2018).

### Polyclonal Human Antibody Produced in Transchromosomic Cattle

Transchromosomic cattle have been developed to produce fully human polyclonal IgG antibodies following immunization (Matsushita et al., 2014). Using this technology, polyclonal sera containing antibodies against the spike protein of MERS-CoV were produced (SAB-301) and demonstrated to be effective in neutralizing the virus *in vitro* and reducing lung virus titers in infected mice (Luke et al., 2016). A phase 1 randomized clinical trial aimed to evaluate the safety and tolerability of SAB-301 was conducted (NCT02788188). Results have been published and showed that the polyclonal human antibody is safe and well tolerated in patients at what could be considered therapeutic doses (Beigel et al., 2018).

### Monoclonal Antibodies Produced in Humanized Mice

Using their VelocImmune mice, Regeneron produced fully human mAbs binding the spike protein of MERS-CoV. Two lead mAb candidates (REGN 3051 and REGN 3048) were further characterized *in vitro* and *in vivo* (Pascal et al., 2015). *In vitro*, both mAbs potently neutralized the MERS-CoV while *in vivo* studies in a mouse model of infection demonstrated that treatment of infected mice caused a reduction in lung virus titers and histopathology (Pascal et al., 2015). A phase 1 clinical trial to evaluate the safety, tolerability, pharmacokinetics (PK), and immunogenicity of single ascending doses of a combination of both mAbs administered IV in healthy adult volunteers is currently underway (NCT03301090).

### Broadly Acting Antivirals

One of the main strategies implemented in the clinics for the treatment of MERS-CoV is the use of broadly acting antivirals although supportive therapy continues to be the primary approach for treatment (Modjarrad, 2016). Antivirals such as interferon and ribavirin that show strong inhibition of MERS-CoV *in vitro* have been used in the clinics with different degrees of success. Ribavirin is a synthetic guanosine nucleoside that interferes with the synthesis of viral mRNA. The best effect has been observed using a combination therapy of interferon (IFN) and ribavirin (Momattin et al., 2013). Different type I interferons including IFN-α2b and IFN-β1b have been used in the management of patients with confirmed MERS-CoV infection (McIntosh, 2017). In a study using macaques, animals were treated with IFN-α2b and ribavirin staring at 8 h after infection with MERS-CoV (Falzarano et al., 2013). The infected animals were euthanized at 72 h post-infection which is the peak of disease in this model. The group that received the combination treatment did not developed overt clinical signs of infection, had reduced levels of pro-inflammatory cytokines, reduction in lung virus titers, and reduction in overall lung pathology compared to the group of macaques that did not receive treatment. Most of the data on the efficacy of this combination therapy are from retrospectives studies (Mo and Fisher, 2016; McIntosh, 2017). In a study comparing infected patients that received this combination therapy in addition to support therapy, it was observed that these patients had a significantly better survival at day 14 post-laboratory confirmation of the infection (70%) compared to the patients that only received the supportive therapy (29%) (Omrani and Shalhoub, 2015). In a case study report of a health worker infected with MERS-CoV, the use of IFN-α2b plus ribavirin was associated with his recovery as well as with a beneficial prophylactic effect in his spouse who only developed a mild respiratory illness (Khalid et al., 2015). IFN-β has also been used in combination therapies for the treatment of MERS-CoV infection (Shalhoub et al., 2015; Arabi et al., 2018). In a retrospective study that analyzed a small cohort of patients with laboratory-confirmed infection, it was observed that treatment with the combination of IFN-β1a and ribavirin did not significantly improve survival (Shalhoub et al., 2015). In a different retrospective study, analysis of the data demonstrated that administration of IFN-β improved survival in treated patients. However, after a multivariate analysis, the positive effect was not significant (Al Ghamdi et al., 2016). Results vary between different retrospective studies and this is in part due to the presence of comorbidities and most importantly the time for treatment initiation (Al-Tawfiq and Memish, 2017), since it has been observed that the window of opportunity is very narrow and only effective when started within the first 48 h of confirmed diagnosis (Momattin et al., 2013; Al-Tawfiq et al., 2014; Widagdo et al., 2017). A case study of a MERS-CoV-infected patient who received ribavirin in combination with lopinavir/ritonavir (Kaletra®), a human immunodeficiency virus antiviral, and IFN-α2a, reported that this regimen was effective to treat the patient (Kim et al., 2016). The patient, a 64-year-old male with different comorbidities, started treatment on day 4 post admission and achieved viral clearance just 6 days after initiation of treatment and finally was discharged from the hospital on day 13 after admission (Kim et al., 2016). In a recent study, a combination of ribavirin and lopinavir/ritonavir was used as a post-exposure prophylaxis in health care workers who had been exposed to MERS-CoV-infected patients and demonstrated a reduction of 40% in the risk of infection (Park et al., 2019). A preclinical study in common marmosets infected with MERS-CoV showed that treatment with a combination of lopinavir/ritonavir and IFN-β1b improved the clinical and pathological outcomes of the infected animals (Chan et al., 2015). A phase 2/3 clinical trial NCT02845843 to determine the feasibility, efficacy, and safety of a combination therapy with lopinavir/ritonavir and IFN-β1b for the treatment of MERS-CoV-infected patients was initiated in 2016. The clinical trial was expected to be concluded in July of 2018; however, no results or activity have been reported.

## Conclusions

This review summarizes the current antivirals available for the treatment of influenza and RSV along with potential new therapies for these respiratory virus infections (Table 1). It also addresses the current therapies employing broad-spectrum antivirals for the treatment of MERS-CoV-infected patients (Table 1). Greater advances have been achieved in the field of influenza antivirals. Currently, three groups of drugs are approved for the treatment of influenza, including the newly approved viral polymerase inhibitor baloxavir marboxil and only one approved for RSV, the polyclonal palivizumab. Although effective, these drugs have limitations such as a reduced window of opportunity, potential to produce drug-resistant viruses, and high cost (Palivizumab). This highlights the need for more and better antiviral drugs to treat these respiratory infections. MERS-CoV recently emerged as a zoonotic respiratory infection and, therefore, no effective antivirals are approved for treatment. The initial approach has been to follow examples of antiviral therapies used during the recent SARS outbreak; however, as described in this review, the approach using broad-spectrum antivirals has not been very successful.

**Table 1:** Novel therapeutics currently under development (clinical trials), for the treatment of influenza virus, respiratory syncytial virus (RSV), and Middle East respiratory syndrome coronavirus (MERS-CoV).

Therapeutic compound	Infectious agent	Mechanism(s) of action	Clinical trial phase	NCT#
CR6261	Influenza	Inhibits the binding of the virus to cell membrane receptor	2a	NCT02371668
CT-P27	Influenza	Inhibits the binding of the virus to cell membrane receptor	2b	NCT03511066 NCT02071914
MEDI8852	Influenza	Inhibits the binding of the virus to cell membrane receptor	2a	NCT03903718 NCT03028909 NCT02603952
MHAA4549A	Influenza	Inhibits the binding of the virus to cell membrane receptor	2	NCT01980966 NCT02623322 NCT02293863
Pimodivir	Influenza	Prevents viral RNA synthesis	3	NCT03381196
Favipiravir	Influenza	Inhibits the activity of the RNA-dependent RNA polymerase	3	NCT02026349 NCT02008344
Fludase	Influenza	Prevents viral entry into cells of the respiratory epithelium	2	NCT01037205
Nitazoxanide	Influenza	Impairs the trafficking of the viral hemagglutinin	3	NCT03336619
RI-001	RSV	Inhibits the binding of the virus to cell membrane receptor	2	NCT00632463
REGN2222	RSV	Viral fusion protein inhibitor	3	NCT02325791
MEDI8897	RSV	Viral fusion protein inhibitor	2	NCT02878330
ALX-0171	RSV	Viral fusion protein inhibitor	2	NCT03418571
131-2G	RSV	Interferes with the attachment process by blocking the G protein	Pre-clinical	NA
Motavizumab	RSV	Viral fusion protein inhibitor	3	NCT00121108 NCT00628303 NCT00129766
GS-5806	RSV	Blocks the virus-cell fusion process	2	NCT01756482 NCT02534350 NCT02254408 NCT02254421 NCT02135614
MDT-637	RSV	Inhibits virus entry to the target cells	1	NCT01489306 NCT01355016 NCT01556607
JNJ-53718678	RSV	Viral fusion protein inhibitor	2	NCT03656510 NCT03379675 NCT02387606
AK-0529	RSV	Inhibits virus entry to the target cells	2	NCT03699202 NCT02654171
TMC353121	RSV	Viral fusion protein inhibitor	Pre-clinical	NA
RSV-60444	RSV	Inhibits RNA synthesis and the infectivity of released viruses	2a	NCT00232635
ALN-RSV01	RSV	Inhibits RNA replication	2	NCT00658086 NCT01065935 NCT00496821
ALS-008176	RSV	Inhibits RSV polymerase	2	NCT02094365 NCT02673476
Convalescent plasma	MERS-CoV	Inhibits virus entry to the target cells	2	NCT02190799
SAB-301	MERS-CoV	Inhibits virus entry to the target cells	1	NCT02788188
REGN 3051 and REGN 3048	MERS-CoV	Inhibits virus entry to the target cells	1	NCT03301090
Ribavirin	MERS-CoV	Interferes with the synthesis of viral mRNA	1	NCT02627378

Great advances have been made in the last few years and several antivirals are currently under development and many have reached early stages in clinical trials. This is a very exciting and evolving field that will continue producing more effective antivirals that are highly needed for the treatment of respiratory viral infections of global public health concern.

## Author Contributions

MB and VL-G wrote, reviewed, and approved the submitted manuscript.

### Conflict of Interest Statement

The authors declare that the research was conducted in the absence of any commercial or financial relationships that could be construed as a potential conflict of interest.
